# Pioneering nanomedicine in orthopedic treatment care: a review of current research and practices

**DOI:** 10.3389/fbioe.2024.1389071

**Published:** 2024-05-27

**Authors:** Wenqing Liang, Chao Zhou, Hongwei Zhang, Juqin Bai, Hengguo Long, Bo Jiang, Lu Liu, Linying Xia, Chanyi Jiang, Hengjian Zhang, Jiayi Zhao

**Affiliations:** ^1^ Department of Orthopaedics, Zhoushan Hospital of Traditional Chinese Medicine Affiliated to Zhejiang Chinese Medical University, Zhoushan, China; ^2^ Department of Orthopedics, Zhoushan Guanghua Hospital, Zhoushan, Zhejiang, China; ^3^ Rehabilitation Department, Zhoushan Hospital of Traditional Chinese Medicine Affiliated to Zhejiang Chinese Medical University, Zhoushan, China; ^4^ Medical Research Center, Zhoushan Hospital of Traditional Chinese Medicine Affiliated to Zhejiang Chinese Medical University, Zhoushan, China; ^5^ Department of Pharmacy, Zhoushan Hospital of Traditional Chinese Medicine Affiliated to Zhejiang Chinese Medical University, Zhoushan, Zhejiang, China

**Keywords:** nanotechnology, nanoparticle, orthopedic, orthopaedic surgery, bone regeneration

## Abstract

A developing use of nanotechnology in medicine involves using nanoparticles to administer drugs, genes, biologicals, or other materials to targeted cell types, such as cancer cells. In healthcare, nanotechnology has brought about revolutionary changes in the treatment of various medical and surgical conditions, including in orthopedic. Its clinical applications in surgery range from developing surgical instruments and suture materials to enhancing imaging techniques, targeted drug delivery, visualization methods, and wound healing procedures. Notably, nanotechnology plays a significant role in preventing, diagnosing, and treating orthopedic disorders, which is crucial for patients’ functional rehabilitation. The integration of nanotechnology improves standards of patient care, fuels research endeavors, facilitates clinical trials, and eventually improves the patient’s quality of life. Looking ahead, nanotechnology holds promise for achieving sustained success in numerous surgical disciplines, including orthopedic surgery, in the years to come. This review aims to focus on the application of nanotechnology in orthopedic surgery, highlighting the recent development and future perspective to bridge the bridge for clinical translation.

## Introduction

Throughout history, innovation and disruptive technology have shown the capability to improve patient outcomes significantly. One field with the potential to revolutionize healthcare is nanotechnology, offering opportunities to enhance the diagnosis and treatment of intricate medical conditions (Chen, 2023; Kia et al., 2023; Malik et al., 2023). Nanotechnology was first described by the National Nanotechnology Initiative as an investigation and controlled manipulation of small molecules and atoms ranging in size from 1 to 100 nm; though, since then, the term has been broadened to incorporate more diverse types of research activities as well as uses (Rambaran and Schirhagl, 2022; Wang et al., 2023). The evolution of nanotechnology in healthcare has marked a revolutionary shift in medical science, offering precise treatments and early diagnosis possibilities. It began with the theoretical foundation laid down by physicist Richard Feynman in his famous 1959 lecture “There’s Plenty of Room at the Bottom,” and gained momentum with the development of the scanning tunneling microscope in the 1980s (Feynman, 2018). This allowed scientists to visualize and manipulate individual atoms. In the early 2000s, the first nanodrug, Doxil, was approved for cancer treatment, showcasing the potential for nanotechnology to enhance drug delivery. Since then, nanotech has continued to break new ground with developments like quantum dots for imaging, nanorobots for surgery, and nanoparticle-based vaccines, including those used in COVID-19. This progression towards increasingly sophisticated nanomaterials and devices is paving the way for more targeted and efficient healthcare solutions (Sullivan et al., 2014).

The need for quick and predictable processes, as well as the need for precise therapies when individuals seek dental treatment (DDS), have prompted several fundamental studies intended for identifying biomaterials with novel features for the dentistry market (Donos et al., 2019; Omar et al., 2019; Eivazzadeh-Keihan et al., 2020; Kunrath et al., 2020). Among these substances, biomaterials with nano-engineered structures and drug delivery systems have demonstrated potential uses, for example, those identified in dental implant oral reintegration (Donos et al., 2019; Kunrath et al., 2020), directed restoration of bone using substitute bones and membranes (Furtos et al., 2017; Donos et al., 2019; Eivazzadeh-Keihan et al., 2020), resins used in dental reconstructive (Imazato et al., 2017) and endodontic products (Zhang et al., 2023a) for intracanal contamination control (Cuppini et al., 2019).

**FIGURE 1:**
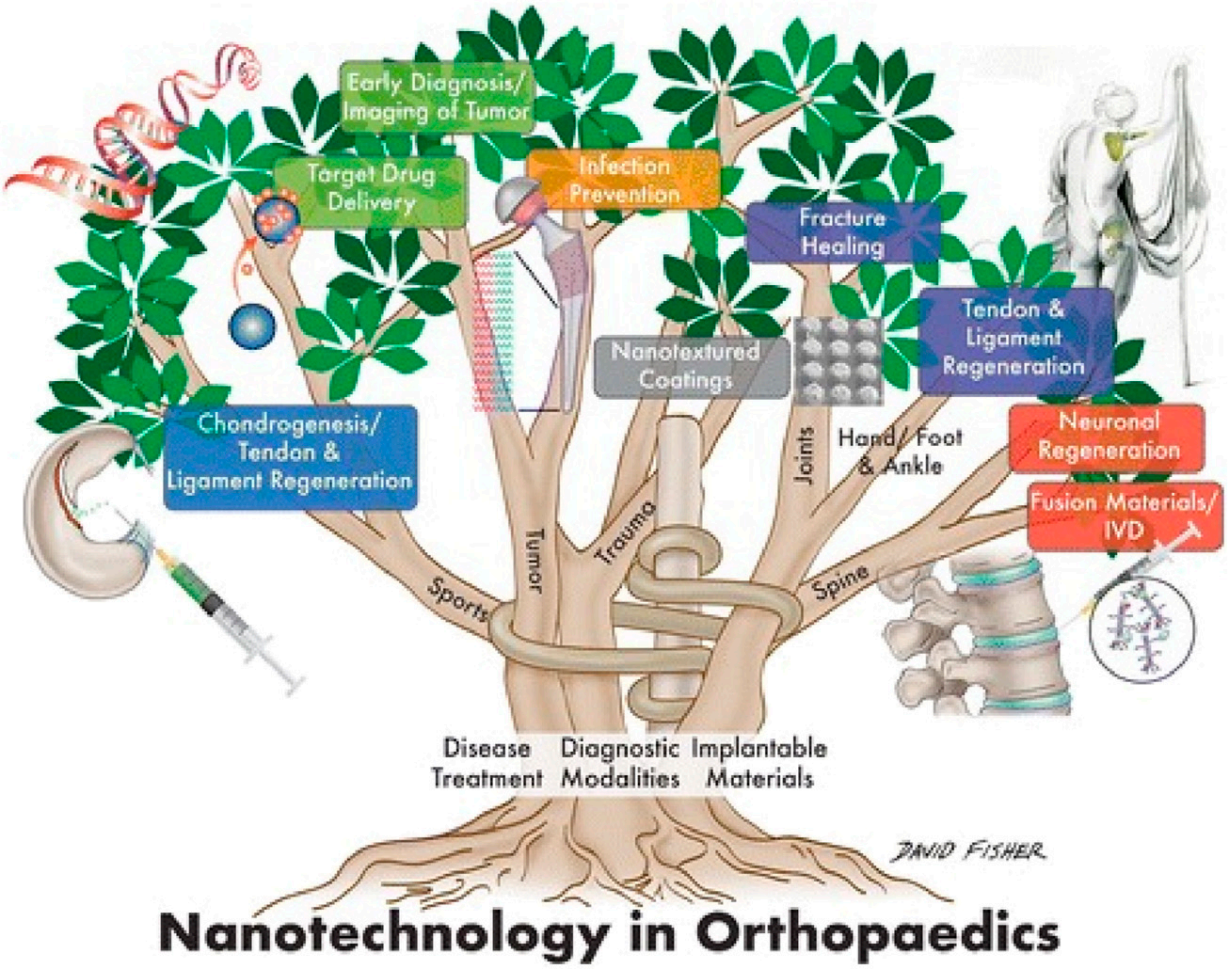
Application of nanotechnology in the orthopedics (Boretto et al., 2023).

Orthopaedics is an attractive field in which nanotechnology can be applied because bone and its constituents, for example, collagen fibrils, hydroxyapatite, and Haversian systems, are nano-compounds (Tasker et al., 2007; Lantieri et al., 2022; Boretto et al., 2023). Bone is composed of a malleable matrix and linked minerals. The matrix of bones is made up of flexible collagen fibers and pulverized material (Zhang et al., 2023b). It consists of a mineral composition of phosphate and calcium in the form of hydroxylapatite (HA), water, and proteins containing type I collagen fibrils. Minerals and organic compounds have nanometer-scale dimensions (Rho et al., 1998). In orthopedics surgery, biomaterials and host tissue commonly interact on a micro-level (Gai et al., 2023). Through nanoscale material alterations, it is feasible to significantly improve the effectiveness of these kinds of interactions by employing biomaterials made up of NPs and structures (Mazaheri et al., 2015; Wang and Tang, 2019). This acts as the basis for the vast majority of orthopaedic applications for nanotechnology. The use of nanotechnology in the orthopaedic investigation is promising since it facilitates the improvement of mechanical features and biocompatibility of implanted orthopedics maneuvers (Han et al., 2022). Nanostructured grafts and prostheses give greater mechanical strength, improved resistance to corrosion and erosion, the ability to administer medicine, and the capacity to act as scaffolds for tissue renovation (Kienapfel et al., 1999; Bishop et al., 2012; Hanc et al., 2016). Nanotechnology offers a vast array of innovative orthopedic applications. Important application includes osseointegration of graft materials, restoration and rejuvenation of meniscus, deformation in the osteochondral, and vertebral disk. It plays an essential function in the delivery of targeted drugs for the cure of bone malignancies (Balasundaram and Webster, 2006; Laurencin et al., 2009; Pleshko et al., 2012; Mazaheri et al., 2015; Poon et al., 2021). Figure 1 shows the various applications of nanotechnology in orthopedics.

In younger patients, conventional orthopedic and dental implants present the greatest concerns of failure and short lifespan. Nanotechnology-created bone substitutes have increased the durability and longevity of implants. Implant manufacturing innovations have enabled the creation and application of biosensors, and diagnostic systems that are sensitive, and controlled DDS (Kon et al., 2009).

Improving implant durability, treating vertebral osteoporotic fractures, controlling infection, orthopedic tissue engineering (TE), treating orthopedic oncology, as well as stem cell rejuvenation medicines are applications of nanomaterial in orthopedic surgery. Due to changes in the physical features and ensuing energies of the original substances, nanomaterials have excellent physiochemical characteristics. The primary applications for implants are (i) replacement of joint reconstruction, (ii) implants in the spine, (iii) orthobiologics, and (iv) implants for trauma (Zhang et al., 2021). This review aims to give an overview of the future of nanomedicine in orthopedics surgeries and will highlight the recent advancement in the surgical procedure using nanotechnology.

## Epidemiology: keeping orthopaedic disorders in perspective

Orthopedic disorders are becoming more predominant in the United States, negatively influencing millions of Americans’ health and leading to increasing healthcare expenses. In 2005, Orthopaedic diseases cost an estimated $849 billion (Brenner and Ling, 2012). With a population of 39.6 million individuals over 65 in 2009 and forecasts that this age group will nearly double to 72.1 million by 2030, the requirement for orthopedic care will rise as the frequency of orthopaedic damage and illness rises with age. In 2004, orthopedic injuries alone contributed to $127.4 billion in medical expenses. This is a forty percent upsurge from 10 years ago. Lost employment contributes significantly to the expense of orthopaedic injuries. A little more than one in ten Americans per year report that they were unable to work due to an orthopaedic injury. Due to musculoskeletal injury, there are also a large number of workers with limitations. In 2004, four out of every 100 individuals and 11 out of every 100 people over the age of 65 had joint dysfunction or bone fracture-related labor limitations (Jacobs et al., 2008).

Arthritis is a chronic condition that impacted approximately 50 million persons in 2003 and led to expenditures of $128 billion. Osteoarthritis, lupus, gout, rheumatoid arthritis, and fibromyalgia are arthritic conditions (Cheng et al., 2010). In 2004, there were 249,000 reported cases of arthritis in minors under 18 years of age in the United States. Arthritis is related to considerable morbidity, with 42% of diagnosed adults reporting physical restrictions as a result of their disorder. Moreover, arthritis is the leading cause of joint replacements. In 2004, there were 232,886 hip substitutes, 454,652 knee replacements, 41,934 shoulder substitutions, and 12,055 other replacements of joints, the majority of which were performed due to osteoarthritis. It is expected that the incidence and incidence of arthritic disorders will rise in the subsequent years due to aging with an emergent number of active patients (Centers for Disease Control and Prevention CDC, 2007).

Even though some orthopaedic disorders are induced by preset risk factors, for example, heredity, there are several modifiable risk factors. These include overexertion, obesity, joint instability, and infection. The majority of these risk factors are modifiable through lifestyle modifications as well as other precautionary measures, however, the advancement of nanomedicine uses will also help to reduce the occurrence and morbidity of orthopaedic disorders.

### Prosthetic replacement of joints using nanotechnology

Osseointegration is the activation of quick new bone production, which firmly anchors grafts placed inside the bone (Oh et al., 2023; Pinotti et al., 2023). The implant’s material qualities and mechanical features of the adjacent bone tissue must be compatible. To accomplish effective osseointegration, they must form direct physical and chemical connections with nearby bone surfaces. There should be no formation of fibrous tissue interfaces. For their excellent mechanical characteristics, cobalt chrome alloys and stainless steel are utilized, but the rigidity of solid materials has led to stress protection and destruction of bones. Osseointegration reduces tension as well as strain at the tissue-implant junction for improved implant efficiency and durability (Dondani et al., 2023; Lang et al., 2023; Sigilião Celles et al., 2023).

**FIGURE 2:**
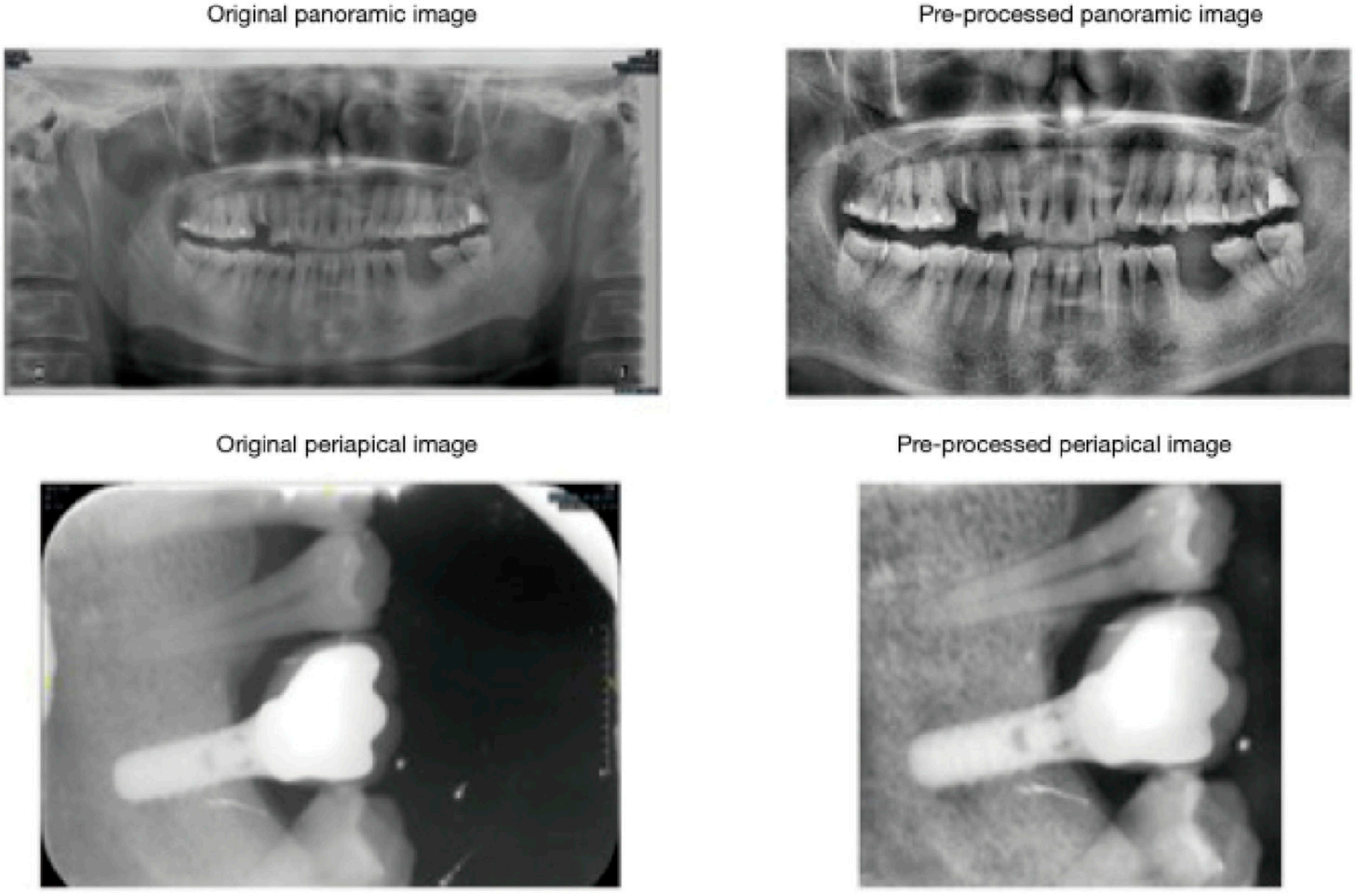
Cropped and histogram-equalized panoramic and periapical images demonstrate successful implant outcomes. Pre-processing steps, including cropping and histogram equalization, were applied to periapical and panoramic radiographs to optimize image contrast. Reproduced with permission from Zhang C. et al. (2023c).

One of the key problems connected with the rising popularity of uncemented complete joint arthroplasties is osteointegration failures (Zhang C. et al., 2023; Verma et al., 2023). Although prosthetic joints are presently cured to improve osseous ingrowth through surface irregularity, the nanoscale, where cellular relationships take place, stays smooth (Figure 2). This stimulates fibrous because of bone ingrowth, leading to early failure (Katz et al., 2013).

Periprosthetic joint infection is one of the primary causes of primary joint replacement failure and modification (Alrayes M. M. and Sukeik M., 2023; Alrayes M. M. and Sukeik M. T., 2023; Patel, 2023). It has been revealed that adherence to bacteria and colonization are reduced. As a result, controlled antibiotic-releasing prosthetic nanophasic joints may offer a viable solution to the catastrophic risk presented by periprosthetic joint infections (Gusić et al., 2014).

Surface nanostructuring with material can be designed to develop active anti-infective surfaces even at an extremely low level of bacterial adhesion. One of the main advantages is greater efficacy, combating a greater variety of bacteria when antibacterial nanoparticles are applied to the surface of titanium. The latter could help reduce the risk of infections in medical implants. Particles could be designed with a targeting ability to destroy bacteria and, at the same time, minimize the risk of developing resistance to bacteria (Cazzola et al., 2023; Gamna et al., 2024). However, there are disadvantages to consider as well. In the long run, the impact on human tissues may turn out to be potentially toxic. Further, nanoparticles may always exist for an extended possibility of time to leach out, hence giving a potentially reduced antibacterial effect, at the same time possibly being harmful to the environment. Integration of nanoparticles into the titanium surface may result in changes in properties such as strength and durability. One must observe such changes in the performance of medical implants. Further studies and developments will need to be done to optimize such surfaces for safety and effectiveness (Cazzola et al., 2023; Gamna et al., 2024).

**FIGURE 3:**
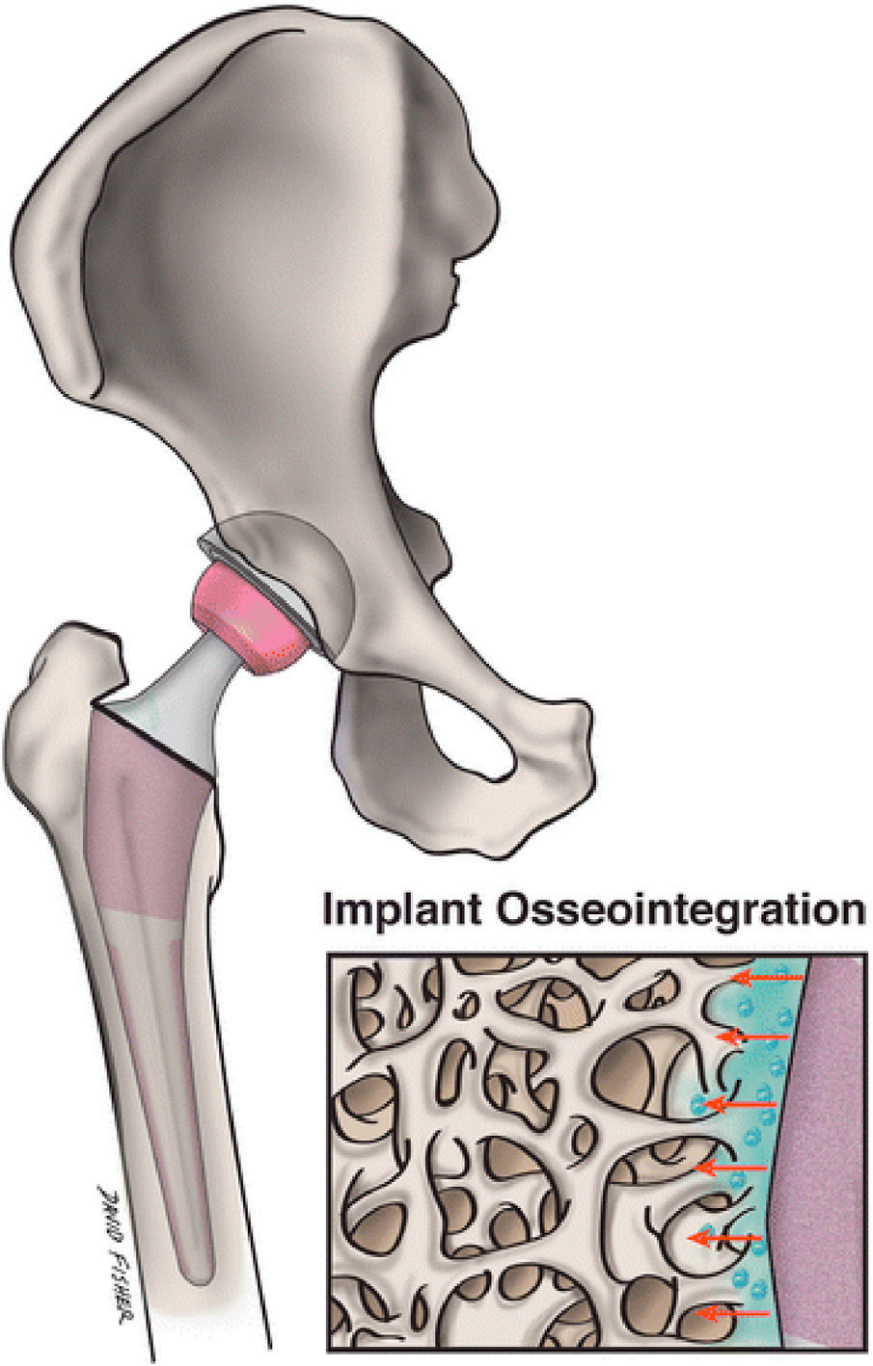
Nanostructured implants may more closely resemble the setting of natural bone and encourage osseointegration of implants and surrounding osteogenesis than traditional grafts. This picture shows the topographical interaction between a nanoengineered graft surface and surrounding bone (Boretto et al., 2023).

Even though primary joint replacement surgery has a high rate of success, its durability is limited. Thin (nano) film coatings may significantly enhance the durability and functionality of artificial joints by providing a robust barrier that minimizes wear and tear through improved friction resistance (Noori et al., 2023). Nanotechnology is used in arthroplasty to target the advancement of materials for implants that are safe to use and efficient while extending the average lifecycle of grafts and averting infection. More favorable contact between the graft and the surrounding bone can be formed by adjusting particular surface features of the graft (Figure 3) (Smith et al., 2018).

### Nanotechnology in the cure of osseous and chondral defects

The treatment of trauma-induced abnormalities of the segmental bones, fixations on failure, and arthroplasty provides a significant challenge (Boretto et al., 2023; Garabano and Pesciallo, 2023). Current strategies for resolving these problems employing auto/allografts and porous metals have limits of their own, for instance, partial availability, infection risk, and insufficient scaffolding elements, which limit the quantity of osteointegration. Since the degree of biomaterial adherence to host tissues determines the optimal scaffold for promoting osteointegration, nanostructured biomaterials are suitable because osteoblasts may colonize them (Andreacchio et al., 2018). Cells may interact, proliferate, and change into natural tissues on the ultimate scaffolds.

Nanostructured biomaterials can provide structural assistance and suitable pore size while also serving as a medium for the movement and activity of cells. When treated with growth factors and chemokines, they can also give biochemical cues to govern tissue change with pharmacological support by transporting peptide patterns that attach to receptors and stimulate intracellular signaling pathways. Nanomaterials with these characteristics are thought to be ideal for treating massive bone deformities (Luthringer et al., 2013; Roddy et al., 2018). Nanoscaffolds can be used to facilitate more natural healing without the complications linked to implants and biomaterials that do not disintegrate since they will eventually resorb after completing their biochemical, structural, biological, and templating roles (Roddy et al., 2018).

**FIGURE 4:**
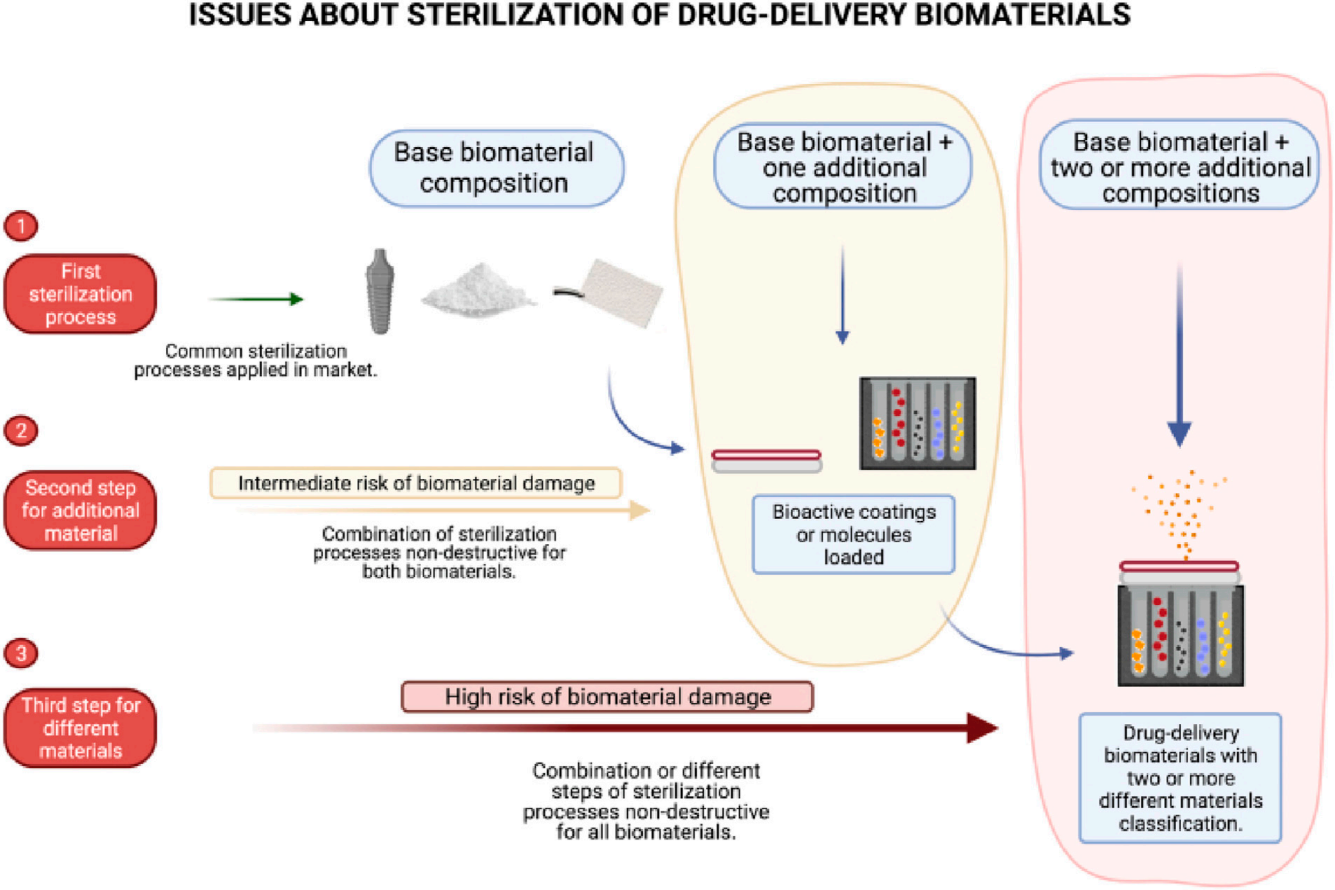
Illustration depicting the challenges encountered in achieving thorough sterilization of biomaterials used in drug delivery. Reproduced with permission from Kunrath et al. (2023).

Numerous natural and artificial nanostructured compounds have been investigated for the management of bone deformities (Wan et al., 2023; Wen et al., 2023). Natural biomaterials have the benefit of being highly biocompatible, however, the way they handle features and support from structures is inadequate. Artificial components, in contrast, give outstanding structural support but are not biocompatible. Presently, artificial biomaterials, for example, hydroxyapatite (HA) and derivatives), bioactive ceramics TCP (tricalcium phosphate) and polymers such as poly-lactic acid (PLA) and poly-glycolic acid (PGA), and a mixture of these, referred to as composite matrices is chosen as scaffolding materials used to cure bone deformities because of their enhanced structural support. Surface cure with growth agents of these nanostructured biomaterials, for instance, bone morphogenic proteins (BMP) and bone sialoproteins (BSP) may enhance their capacity to osseointegrate effectively. Natural polymers which include gelatin and fibrin have also been used to repair non-load-bearing bone deformities such as cranial abnormalities (Vasita and Katti, 2006; Bakhori et al., 2023; Krishani et al., 2023). The challenge lies in devising a sterilization protocol for Biomaterial-Based Drug Delivery Systems (BDDS) that incorporate multiple types of base biomaterials, such as combinations involving metals with drugs, metals with molecules, metals with polymers, and polymers with molecules, among other permutations (Figure 4).

However, this revolution in the use of BMP2 to improve bone regeneration does come along with several potential risks, among them being that of inducing abnormal growth of bones (Halloran et al., 2020). This can result in clinical complications, one of which is the undesired formation of bone at the sites nearer to the area of treatment. To have that happen, it would have to be discarded from the area where bone growth is not intended (James et al., 2016). Besides BMP2, a larger class of nanomaterials used for medical interventions carries a number; for example, certain cytotoxic effects of the nanomaterials are likely to bring an adverse response to cells. The high development and implementation cost of nanotechnology remains one of the principal barriers to wide-scale in any sphere of its utilization. Unpredictable results of nanomaterials partially engendered by their complex interplay with biological systems require a more precautionary attitude (Zara et al., 2011). This further underscores the real possibility that current This only indicates further the urgency of very thorough research and regulation that, in some way, would limit the mentioned risks and secure these technologically advanced medical treatments in the best possible way.

Cartilage has a more convoluted structure, making the cure of cartilaginous disorders more complex employing biocompatible or artificial scaffolds. Due to their enhanced capacity for biodegradation, cell infiltration, biocompatibility, and neovascularization (Vasita and Katti, 2006), biological protein scaffolds, for example, collagen and polysaccharide scaffolds for instance hyaluronic acid, chondroitin sulfate, chitosan, and agarose are recommended therapies for cartilage abnormalities. Regardless of their immunoreactivity, type I collagen frameworks are the most frequent. In individuals with chondral defects, acid-treated collagen polymers comprising mesenchymal stem cells (MSC) have been suggested to create hyaline-like cartilage. The denatured form of gelatin is an alternative to collagen that is immune-reactive and ailment-transmissible (Banimohamad-Shotorbani et al., 2023; Jeyaraman et al., 2023).

Since most cartilage abnormalities are manageable and less invasive operational measures, the availability of injectable frameworks is serious. Hydrogels are injectable nanoscale polymeric networks made up of gelatin or collagen, with the capability to consolidate and take on the required form of the issue after embedding. When hydrogels are injected with chondrocytes, they form cartilage-like ECM with increasing mechanical improvement as a result of the ongoing formation of a glycosaminoglycan-rich matrix (Ahmadian et al., 2023; Guo et al., 2023; Stone et al., 2023).

Applying nanofibers to make osteogenic or chondrogenic scaffolds has shown several advantages, including increased propagation, cell adherence, and movement. Nanofiber scaffolds had the largest concentration of type II collagen, an enhanced capability to absorb human blood proteins, as well as a substantial increase in the expression of cartilage-specific genes and proteins, i.e., collagen II and IX. Several reported studies have shown that TE for the management of cartilage and osseous deformities is one of nanotechnology’s most important applications and related studies in orthopaedics (D'Antimo et al., 2017).

## Materials for bone restoration

**FIGURE 5:**
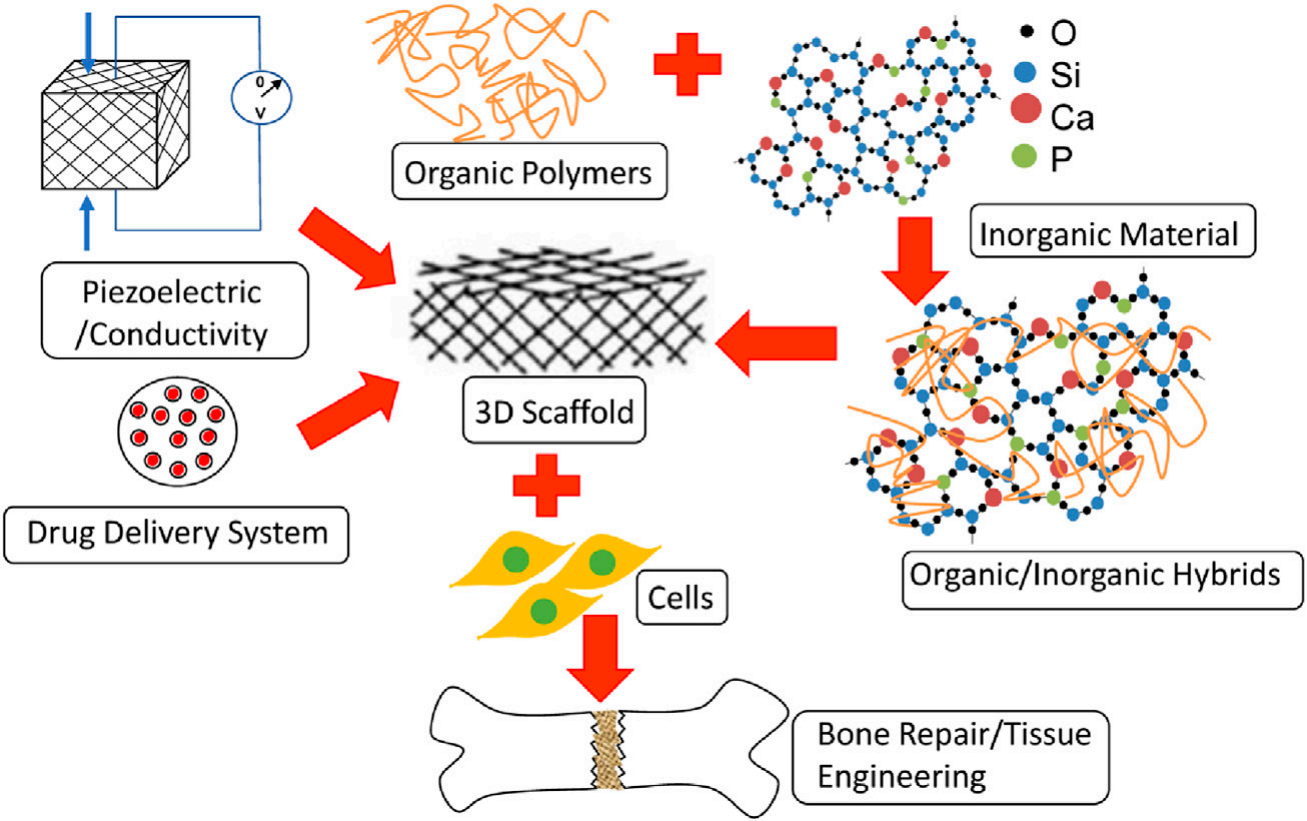
Application of various materials in bone repairs and regeneration (Aslankoohi et al., 2019).

Synthetic bone is a bone-like substance developed in a laboratory and used as a bone graft. Bone is composed primarily of hydroxyapatite crystals and collagen fibers. Also present are keratan sulfate, chondroitin sulfate, and lipids. In bone grafting procedures, organic polysaccharides (chitosan, chitin, and alginate) as well as minerals (hydroxyapatite) are created as materials. Bone cement supplemented with nanoclay possesses improved mechanical characteristics. Nanophase characteristics are present in alumina, selenium, titania, nanoceramics, cobalt chrome alloys, carbon, Ti6AlV, nanometals, and nanocrystalline diamond (Sato and Webster, 2004; Durmus and Webster, 2012) Bone substitute substances are utilized for treating bone fractures, hip revision periprosthetic fractures surgeries, spinal column surgery cage filling, reconstruction of the acetabulum, osteotomies, and bone abnormalities in children (Arts et al., 2006). The purpose of hybrid bone biomaterial is to replicate the composition of native bone, these materials are designed to supplant. Together with dimensional and mechanical integrity, scaffolds for bone renovation must provide cells with suitable microenvironments. Consequently, scaffolds function as more than just a basic framework, indicating the need for more effective and interactive biomaterials (Figure 5); (Aslankoohi et al., 2019).

### Application characteristics of bone substitution components

Injectable bone matrix composed entirely of nanoparticle-sized hydroxyapatite. After a few months, this is fully assimilated (Appleford et al., 2009). Engineered artificial bone items with bone void filler consisting of HA nanocrystals that have a structure similar to natural bone crystals.

Tricalcium phosphate nanoparticles have been devised as a substitute substance for bone with cancellations prostheses. When contrasting with conventional tricalcium phosphate, their porosity, surface area, vascular invasion, and bioresorption are all increased (Szpalski and Gunzburg, 2002).

Nanocomposite scaffold grafts consisting of nanostructured HA and Type I collagen are employed to repair osteochondral abnormalities in the knee joint.

### Nanotechnology for osteoporosis prevention and therapy

Osteoporosis is defined by a decrease in bone mass and micro-structural bone injury. It could be either primary or secondary (Adami et al., 2022; Głuszko et al., 2023; Qaseem et al., 2023). Due to osteoporosis, spinal fractures are more prevalent than other types of bone fractures. The goals of osteoporotic vertebral fracture (OVF) treatment are pain relief, restoration of height, as well as the functional integrity of the affected vertebral body. The nanosized bioavailability of calcium citrate and calcium carbonate is improved via nanotechnology, lowers the risk of osteoporosis, and is employed to treat osteoporotic vertebral fractures. Bone fillings, nanomaterials that can be injected, and Polymethyl-methacrylate (PMMA) bone cement can be utilized to perform vertebroplasty and kyphoplasty. With the improvement of calcium sulfate cement (CSC), and calcium phosphate cement (CPC) (Barinov and Komlev, 2011; Świeczko-Żurek et al., 2022), the clinical applications of bone cement have improved. Hydrogels that can be injected are innovative instruments for bone regeneration and healing (Cheng et al., 2023; Li et al., 2023).

A perfect example of injectable NPs for kyphoplasty and vertebroplasty must have good injectability and uniformity during injection. It must have setting characteristics with adequate handling periods. It must have sufficient mechanical strength and rigidity to correspond with neighboring vertebral bodies (Arora et al., 2013). They must provide porous structures for osseointegration and angiogenesis, as well as optimal stimuli for new bone formation. It should be free of necrosis and infection, as well as radiopacity for surgical imaging. Conventional components have monomer toxicity, increased temperatures that cause tissue injury, an inability to incorporate into bone, and severe stiffness that leads to fracture. New bone cement incorporating nanomaterials eliminates these problems. The osteoblast adhesion densities of PMMA bone cement incorporating nano-phase BaSO_4_ and MgO are greater than those of PMMA cement alone (Ricker et al., 2008; Karpiński et al., 2019).

The use of CPCs in bone deformities and TE. It is chemically and biologically comparable to normal bone, can be shaped after combining, and is a substitute for PMMA bone cement. CPCs are categorized as either brushite (dihydrate of dicalcium phosphate) or apatite. CPC ultrafine nanofibers, akin to cortical bone, improve fracture resistance. Pores and interconnecting channels for bone ingrowth are created by fiber degradation (Canal and Ginebra, 2011) When PMMA is blended with a 2% aqueous gel solution of sodium hyaluronate, its elastic modulus and yield strength decrease (Liang et al., 2024). The addition of CNTs to CPC increases its strength, as does the bio-mineralization of CNTs (Wang et al., 2007). The combination of bovine serum albumin (BSA) and multiwalled CNTs results in a CPC with superior strength (Chew et al., 2011). The CPC/multi-walled CNT/BSA composite enhances the material’s strength and interface bonding with CPC, as well as its wettability and reactivity. Further, BSA and MWCNT enhance the mechanical characteristics of CPC composites, resulting in stronger materials, and encouraging HA development. Current CPC uses include three-dimensional printing, stem cells, injectability, drug delivery, and growth factor. Their uses comprise prefabricated CPC scaffolds, injectable CPC scaffolds, 3-dimensional printing, and CPC scaffold assembly for bone TE.

### Bone regeneration

**FIGURE 6:**
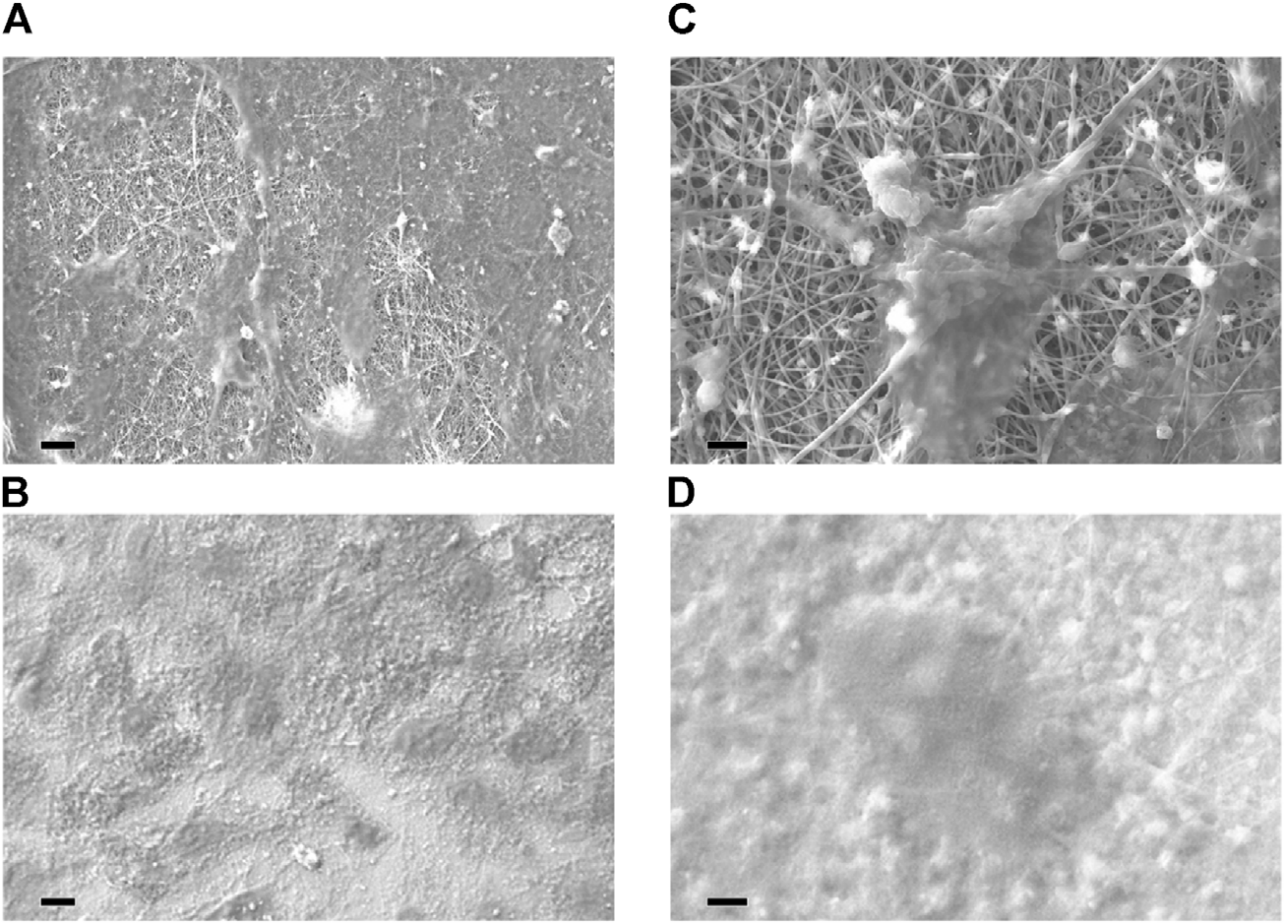
SEM analysis of cell adhesion to distinct scaffolds. Both nanofibrous scaffold **(A,C)** and control film **(B,D)** supported cell adhesion. At increased magnification, a single cell is shown to be firmly affixed to and distributed across the electrospun nanofibers **(C)**, with which it is intimately associated. Cells seeded on control films exhibited a lesser degree of dissemination **(D)**. Comparable pictures were acquired from PCL-HA samples, PCL, and PCL-TCP samples. 10 m **(A,B)** or 2 m **(C,D)** for the bar (Polini et al., 2011).

Similar to collagen, functionalized SWCNTs serve as frameworks for bone treatment (Zhao et al., 2011). Functionalized SWCNTs are optimal for creating synthetic bone and promoting bone development. They act as a scaffold made for the nucleation of collagen and production of HA in bone when inserted as solutions or scaffolding as a substratum (Zhao et al., 2005) When human mesenchymal stem cells (hMSC) are incubated on TiO_2_, tiny nanotubes readily acquire local proteins and generate an ECM-like environment that facilitates hMSC adhesion. Larger nanotubes elongate human mesenchymal stem cells and cause them to differentiate into osteoblastic cell lines (Oh et al., 2009). Greater nanotubes acquire fewer local proteins, and human mesenchymal stem cells (hMSCs) create filopodia to cover a broader surface area and ensure optimal adherence. This method enhances osteoinduction involving gene treatment (Figure 6) (Bhakta et al., 2005; Polini et al., 2011).

Nanostructures designed for orthopedic treatments often exhibit unique surface morphologies, such as high porosity or specific topographies that encourage bone cell adherence and proliferation, thereby aiding in bone tissue integration (Liang et al., 2024). The internal structure, potentially visible through high-resolution imaging, might include a strategic arrangement of pores or channels that supports nutrient flow and waste removal, mimicking natural bone architecture. These features collectively work to enhance the mechanical properties of implants, ensure biocompatibility, and improve patient outcomes by accelerating the healing process and reducing the likelihood of implant rejection (Chen et al., 2023).

### Orthopaedic surgical stem cell regenerative medicine

Loss of tissues or organs is caused by old age, illness, injury, and certain genetic defects. The method of repairing fractionally depleted tissues is known as regenerative medicine. Regenerative medicine permits the cultivation of tissues in the laboratory, their safe implantation, and regeneration. Stem cells possess a vast capacity to repair themselves and have numerous regenerative medicine applications. The development of biomaterials is another advance in regenerative medicine. Surface nanopatterning alters some biological reactions of host tissues, whereas nanotechnology supplies nanomaterials and scaffolds for TE (Pal et al., 2011).

### Nanotechnology in imaging during orthopedic surgery

Nanotechnology has increased to the top of the imaging business during the last 10 years. The area of orthopaedic surgery, which depends substantially on imaging technology, stands to benefit greatly from advances in the area. The semiconductor particles are known as quantum dots with a diameter of 2–10 nm that produce photons specific to the site imaging abilities. Quantum dots are valuable because they release particles when stimulated. The photon wavelength produced upon activation is exclusively regulated by the size of the dot, which can be accurately modified (Brenner and Ling, 2012). R-affixed self-illuminating quantum nanomaterials. Reniformis luciferase (RLuc) (So et al., 2006) is of special importance in orthopaedic surgery (Xing et al., 2008; Smith et al., 2009).

Certain applications need a higher spatial resolution to appropriately recognize abnormal properties, although most orthopaedic imaging applications concentrate on macroscale distinction. Osteoporosis, the most predominant degenerative disease in the West, is on top of the list because it needs in-depth imaging to determine the density and morphology of afflicted bone. At present, standard computed tomography (CT) has a resolving power of slightly around 1 mm, which is too big to view microscopic bone characteristics for instance osteocyte lacunae and canaliculi that link them. Quantitative imaging is made possible by a new technology that uses ptychographic CT to construct 3D density studies having a resolution of less than a micron. In contrast to standard lenses, ptychography is predicated on refractive microscopy. It makes use of sensors with fast speeds to gather micro-diffraction patterns created when electromagnetic (EM) radiation (X-ray in this example) contacts the sample (Dierolf et al., 2010).

Magnetic resonance imaging (MRI) is another modality that will be enhanced by nanotechnology. Sykova and others were able to track cellular movement using images of T2-weighted MRI (Sykova and Jendelova, 2007) by labeling embryonic stem cells (ESCs) and bone marrow MSCs with superparamagnetic iron oxide (SPIO) nanoparticles. They were able to effectively trace cellular movement in models with cortical and spinal lesions using this visualization system. The ability to employ tracers to pinpoint the exact position of brain lesions implies that future site-specific therapies may be possible.

## Nanotechnology’s role in orthopedic surgery sensors

Making clinical decisions and treatment interventions now rely mainly on sensor technology. Using nanotechnology, developments in the specificity and sensitivity of sensors are increasing. One of the primary causes of orthopaedic failure of grafts is the separation prosthetic stem originates from the bone. Under physiological circumstances, a weight-bearing bone’s strain typically lies in a typical range in terms of age and location. Bone tumors, the progression of osteoporosis, as well as prosthetic integration are investigated via *in vivo* measures of strain that leave usual boundaries. Metallic strain gauges made of foil are currently commercially available, but their lack of sensibility, big size, and lack of long-term biocompatibility are constraints. Current strain gauges are being substituted with strain gauges that have been upgraded in every manner by nanotechnology (Alpuim et al., 2008). A network of these microsensors would make it possible to a 3D examination of force-loading capabilities in real-time.

Employing multi-walled carbon nanotubes (MWCNTs) developed in cavities on a titanium surface to measure the amount of *in situ* bone development is an alternative technique for sensing *in situ* bone progression. MWCNTs work by finding the relative resistivity of emerging on the graft; bone HA is conductive, while microbes and scar tissue have an elevated level of resistance (Liu et al., 2007; Brenner and Ling, 2012). Furthermore, when compared to titanium grafts already in use, These MWCNTs promote the calcium buildup of osteoblasts (bone-forming cells) (Sirivisoot and Webster, 2008).

Existing *in vivo* sensors have limitations because they are incapable of being powered for lengthy periods. The Lajnef investigative team intends to overcome this constraint through the use of ultralow power (1 lW) piezo strain gauges that are also capable of collecting energy (Lajnef et al., 2008), thereby permitting the implant sensors to continue to function continuously. Also under investigation (Liu et al., 2007) is the ability to wirelessly send growth and strain data to an external receiver using shortwave radio (Bluetooth) and radiofrequency identification (RFID), allowing the data to be used successfully while making clinical decisions.

### Orthopedic implantable nanomaterials

**FIGURE 7:**
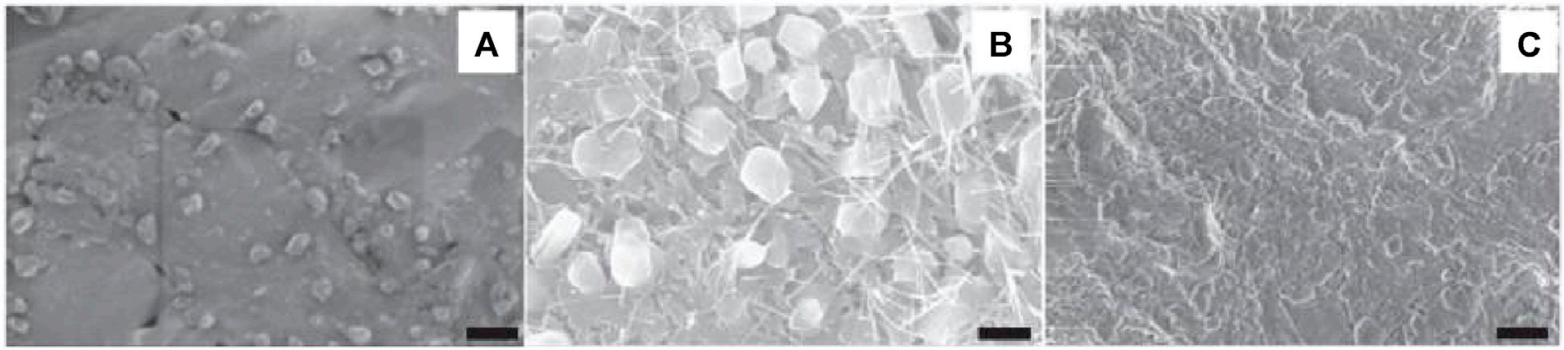
**(A)** SEM images of an untreated selenium compact (SC). **(B,C)** SC treated with 1N NaOH for 10 min and 30 min, respectively. Reproduced with permission from Tran and Webster (2008).

Implantable biomaterials include magnesium alloys, stainless steel alloys, Ti alloys, cobalt-chrome alloys, alumina, HA, zirconia, carbon fiber/polyetherether-ketone, poly(lactic acid) (PLA), polymethylmethacrylate (PMMA), and carbon fiber/ultra-high molecular weight polyethylene are common materials that can be used to replace bone structurally (Pishbin et al., 2013; Liu et al., 2014; Pompa et al., 2015). Nanostructured materials have become innovative orthopedic implants with better potential for osseointegration as a result of recent advancements in nanotechnology while in contrast to conventional materials, they possess cell-favorable surface characteristics that effectively promote the formation of new bone (Zhang and Webster, 2009). For example, metallic implanted devices that have been nanostructured have improved mechanical and biocompatibility characteristics (Mishnaevsky et al., 2014). Currently, powder metallurgy (P/M) (Mishnaevsky et al., 2014) and severe plastic deformation (SPD) (Serra et al., 2013) methods may be used to economically produce bulk nanocrystalline (NC; <100 nm) and ultrafine-grained (UFG; ∼100–500 nm) metals, containing Ti and related alloys. In this case, strong plastic strains with complicated stress states are applied to bulk metal or powder materials, which causes the coarse grains to break down into the nanoscale range. While having a greater strength (>1,000 MPa) than traditional implants, SPD’s nanostructured titanium implants are bioinert and free of any possible harmful or allergic reactions from alloying elements like Al and V (Serra et al., 2013). A recent research by Gain et al. (Gain et al., 2015), has demonstrated that UFG/NC P/M Ti grafts exhibit superior strength and ductility compared to standard Ti–6Al–4V alloys and SPD-processed Ti components. UFG Ti (170–200 nm) was produced by Estrin et al. (Estrin et al., 2011), using equal channel angular pressing (ECAP) and contrasted the way that a coarse-grained (CG) Ti specimen (4.5 μm) adhered to the surface of hMSCs. It was revealed that there were improvements in the attachment and dissemination of hMSCs within the first 24 h of culture. TiN-coated UFG Ti (∼130 nm) was created by Wang et al. (Wang et al., 2013), using a high-pressure torsion process. The produced material’s strong strength, acceptable ductility, good fatigue life, outstanding abrasion resistance, and harmless ion release show its significant potential as an implant. Park et al. (Park et al., 2009), examined the *in vitro* biocompatibility of UFG Ti generated by ECAP utilizing MC3T3-E1 cells in comparison to Ti–6Al–4V alloy and commercially pure (CP) Ti. The samples have micro-rough surfaces created by grit-blasting them with HA particles. Alkaline phosphatase (ALP) activity, adhesion, osteocalcin, and osteopontin mRNA levels in growing cells, cell spreading, vitality, and mineralization nodule formation were among the improved biological responses shown by the UFG material. Element selenium is another substance used in orthopedics that may have anticancer properties. Selenium, as opposed to titanium, is a necessary trace element in human body. Mammalian selenoproteins, which are involved in thyroid hormone metabolism, antioxidant defense mechanisms, and redox regulation of cell processes, make selenium an essential element (Perla and Webster, 2005). The development of several malignant cell lines has been demonstrated to be inhibited by selenium *in vitro* study (Tran et al., 2010). Selenium has been shown by Perla and Webster (Perla and Webster, 2005) to positively impact osteoblast development. In order to develop roughness on selenium compacts with a nanostructure for chemotherapeutic orthopedic uses, Tran and Webster (Tran and Webster, 2008) shown that a higher level of nanometer-scale selenium roughness enhanced the adherence of healthy bone cells. But because selenium is a metalloid and lacks sufficient mechanical strength, this method of adding selenium may leave the implant with weak or inappropriate mechanical qualities (Figure 7) (Tran and Webster, 2008). Furthermore, stability and control over selenium’s release would be highly desired qualities given that it is poisonous in excessive amounts (Tran et al., 2010). Tran et al. (Tran et al., 2010), have produced a nano selenium-coated Ti to enhance orthopedic applications as an alternate method of employing selenium as an antitumor orthopedic material. Selenium nanoclusters’ potential as a covering for Ti orthopedic materials that can prevent cancer and support the normal functioning of bone cells has been shown. The hardest materials to work with in orthopedic applications include bioceramics, although their innate brittleness prohibited their use in particular applications. Improved fracture toughness and the potential to support biofunctionality are two benefits that nanophased ceramics may provide (Catledge et al., 2002). The nanostructuring of several bioceramics, such as alumina, titania, calcium phosphates, zirconia, bioactive glass (BG), and HA, is one of the most recent developments (Simchi et al., 2011). Research has indicated that nanostructuring results in increased mechanical strength together with enhanced ductility and toughness because the smaller grains prevent dislocation slip and blunt cracks (Ovid’ko and Sheinerman, 2011). Furthermore, when the sintering activity increases, manufacturing nanoceramics at reduced temperatures becomes possible (Simchi et al., 2011). In the meanwhile, it might be difficult to limit grain expansion during high-temperature processing. Additionally, nanophased bioceramics work better with cells *in vivo* and *in vitro*. When it comes to rabbit MSC cell survival and propagation *in vitro*, Zhou et al. (Zhou et al., 2015), have found that NC HA offers a superior substrate than CG HA. Enhanced apoptosis of bone-like HA nanocrystals modified with alendronate has been seen in osteoclast-like cells demonstrated *in vitro* by Bosco et al. (Bosco et al., 2015). In a critical-sized malfunction rabbit ulnar model, effective crack bridging is achieved using nanostructured BG scaffolds directing bone growth (Hafezi et al., 2012). The usage of nanotextured surface and nanoengineered grafts will support in fixing the problem by increasing activity of osteoblastic cells. The increased surface area of nanoengineered grafts allowed greater interaction between the host bone and the graft surface, opening the way to dependable and anticipated osteointegration, thereby lengthening the lifespan of implants. (Guarino et al., 2019; Abaszadeh et al., 2023).

Further, options for bone and potential uses for cartilage tissue engineering include artificial and natural polymers. Biocompatible and physiologically active natural polymers that support cell adhesion and growth include fibrin, collagen, chitosan (CS), HA, and alginate (Agarwal and García, 2015). These natural substances are superior to synthetic ones in sharing similarities with bodily materials and might be employed as scaffolding for the surface of implants (Park et al., 2007; Gallo et al., 2014). Recent developments in the creation of CS-based scaffolds with improved bone redevelopment potential were highlighted by Zhang and Levengood (Streicher et al., 2007). Mandal et al. (Mandal et al., 2012) created composite matrices bonded with silk fibers for use in bone engineering that have a high compressive strength (∼13 MPa in a hydrated condition). To facilitate bone regeneration nanomedicine, Schiavi et al. (Schiavi et al., 2015) created novel collagen nanofiber implants that are personalized with growth factor BMP-7 nanoreservoirs and fortified with MSCs from humans. Additionally, a wide variety of polymer nanofibers have been researched for use in replacing bone tissue (Streicher et al., 2007). Using methods including phase disparity, particle leaching, electrospinning, 3D printing and chemical etching, these nanofibrous or nanoporous polymer matrices may be created (Zhang and Webster, 2009). To investigate the survival, propagation, and differentiation of hMSCs along with their derivatives that are chondrogenic and osteogenic, Xin et al. (Xin et al., 2007) synthesized electrospun PLGA nanofibrous scaffolds. Findings showed that during a 2-week incubation period in PLGA nanofibers, hMSCs consistently differentiated into osteogenic and chondrogenic cells. Improved chondrocyte activities on nanostructured 3D PLGA scaffolds were reported by Park et al., (Park et al., 2005).

Generally speaking, nanopolymers and nanoceramics are employed primarily as coating component material in orthopedic or can be mixed with other biomaterials to create nanocomposites appropriate for use in implants. Since bone is a real nanocomposite, as was previously established, nanocomposites are more advantageous compared to alternative nanostructured materials. Commonly used nanocomposites for the regeneration of bone tissue include carbonaceous nanophases in ceramic or polymer matrix, ceramic nanophases in ceramic matrix, and ceramic nanophases in polymer matrix (Yang et al., 2011; Garmendia et al., 2013). When porous HA/ZrO_2_ nanocomposites were created using the P/M approach, as demonstrated by Gain et al. (Gain et al., 2015). The reinforcing action of ZrO_2_ nanoparticles (NPs) allowed the nanocomposites to display superior compressive strength and elastic modulus compared to porous monolithic HA. A growing number of researchers are interested in using ceramic-polymer nanocomposites as materials for bone tissue regeneration because of the extraordinary fusion of osteoconductivity and bioactivity in ceramics and flexibility and form controllability in polymers (Sahoo et al., 2013). Hickey et al. (Hickey et al., 2015), have created PLLA-based nanocomposites reinforced with MgO NPs and HA very recently. According to their findings, MgO NPs considerably improve osteoblast adhesion and propagation on HA–PLLA nanocomposites while preserving mechanical characteristics appropriate for applications involving cancellous bone. For the medical management of cancellous bone deformities or orthopedic applications with limited load bearing, Sadat-Shojai et al. (Sadat-Shojai et al., 2015) created 3D HA/gelatin hydrogel nanocomposites with increased rigidity. The incorporation of MC3T3-E1 cells into the nanocomposites demonstrated that the bone cells were compatible with the whole composite manufacturing process. Carbon nanoparticles are new, substitute reinforcing materials. The mechanical characteristics of orthopedic materials can be effectively enhanced by the addition of carbon nanostructures, such as graphene, carbon nanofibers, carbon nanotubes, nanodiamond (ND), and so on, because of their very high mechanical strength compared to most other materials (Yang et al., 2011). By employing a hydrothermal method, Baradaran et al. (Baradaran et al., 2014), created composites of graphene oxide (rGO) reinforced graphene (HA) nanotubes. They demonstrated that increasing the rGO concentration enhanced the sintered specimens’ elastic modulus and fracture toughness. Additionally, there were reports of increased osteoblast adhesion and propagation. Wu et al. (Wu et al., 2013), examined the biomimetic development behavior of HA on carboxylic group-customized CNFs and assessed the composites’ fracture strength and structure. The enhanced mechanical strength and ability for HA to form an interfacial link with host tissues were achieved as a result of the strong interfacial interaction between HA and CNFs. Another research investigation (Liao et al., 2013) created biocomposites for bone replacements by combining HA nanorods and multiwalled carbon nanotubes (MWCNTs) with polypropylene. The 3-(4,5-Dimethylthiazol-2-Yl)-2,5-Diphenyltetrazolium Bromide (MTT) assay and mechanical testing indicate that the mechanical properties—like impact toughness, tensile strength, and stiffness—were enhanced without significantly affecting biocompatibility.

### Future direction

Nanotechnology has made a significant impact in medicine, and substantial financing is being dedicated to nanomedicine investigation. Most effective *in vitro* and laboratory-based studies have yet to be converted in clinical settings, despite the reality that the theoretical benefits of nanotechnology are exceptional. There are concerns in relation to the toxicity of NPs produced as abrasion detritus. At the nanoscale, metals exhibit distinct behaviors and material qualities than at the microscale. The small metal ion particles have made havoc with metal-on-metal (MOM) hip substitutions. Therefore, traditional grafts cured by nanotechnology for exact characteristics are preferable over nanoparticle implants. This prevents nanoparticles from becoming dispersed and causing tissue toxicity. Given these reservations, it has been suggested that regulation is required. Industries are still unwilling to make nanostructured grafts and prostheses due to unproven healing advantages, probable toxicity risks, and prohibitively high prices. In conclusion, we consider that nanotechnology progressions will continue to influence medicine’s future, specifically orthopedics. To achieve the prospective clinical benefits, additional investigation is required, which requires regulated regulation in the absence of impeding research avenues.

## Conclusion

Applications of nanotechnology in medicine are transforming disease prevention, diagnosis, and therapy, and will have a profound impact on healthcare in the coming years. Due to the expanding prevalence of orthopedic conditions in the United States, it is crucial to develop and implement nanotechnology-enabled medical interventions that target orthopedic conditions. The nanotechnology applications stated above are only a small part of the ongoing initiatives to design innovative clinical tools for orthopedic surgeons to treat common conditions. Product development and commercialization will be staggered, with the rate at which individual products reach the market dependent on regulatory, economic, health and safety, and other variables. The application of nanotechnology to the diagnosis, prevention, and management of orthopaedic disorders holds considerable potential for enhancing orthopedic surgery care in the twenty-first century.
